# Prospecting the biodiversity of the fungal family Ustilaginaceae for the production of value-added chemicals

**DOI:** 10.1186/s40694-014-0002-y

**Published:** 2014-11-01

**Authors:** Elena Geiser, Vincent Wiebach, Nick Wierckx, Lars M Blank

**Affiliations:** Chair of Applied Microbiology, iAMB – Institute of Applied Microbiology, ABBt – Aachen Biology and Biotechnology, Worringerweg 1, Aachen, D-52074 Germany

**Keywords:** Ustilago maydis, Ustilaginaceae, Organic acid, Polyole, Glycolipid, Itaconate, Malate, Succinate, Erythritol

## Abstract

**Background:**

Ustilaginaceae (belonging to the smut fungi) are commonly known for their plant pathogenicity. Although these microbes lead to yield reduction of cereal production, they can also have an economically positive side. Ustilaginaceae naturally produce a versatile range of value-added chemicals with potential applications in the food, pharmaceutical, and chemical industry.

**Results:**

In this study 68 Ustilaginaceae of 13 species were screened for the production of organic acids, polyols, and glycolipids from glucose to characterize their biodiversity and identify potential novel strains for biocatalysis of these valuable chemicals. *Ustilago cynodontis*, *Ustilago maydis*, *Ustilago avenae,* and *Sporisorium exsertum* were identified as promising production organisms for itaconate, malate, succinate, and erythritol, respectively. The influence of buffer concentration (pH) on acid production was investigated. Selected strains with best itaconate and malate production were characterized in more detail in bioreactor experiments obtaining total acid concentrations of up to 47 ± 1 g L^−1^.

**Conclusion:**

The identification and detailed characterization of these producers of valuable chemicals highlights the potential of these unicellular smut fungi for industrial applications and is a further step towards the biotechnological utilization of Ustilaginaceae.

**Electronic supplementary material:**

The online version of this article (doi:10.1186/s40694-014-0002-y) contains supplementary material, which is available to authorized users.

## Background

The family Ustilaginaceae belongs to the order Ustilaginomycetes (true smut fungi) and contains 17 genera, such as *Macalpinomyces*, *Sporisorium*, *Ustanciosporium*, *Pseudozyma,* and *Ustilago* [1]. The entire family has described 607 species, including the model organism *Ustilago maydis*, which is mostly studied in relation to its plant pathogenicity. Members of the Ustilaginaceae can infect economically important crops including corn, barley, wheat, oats, sorghum, sugarcane, and forage grasses [2]. Symptoms they cause are tumor formation (*Ustilago maydis*) and phyllody in the inflorescences (*Sporisorium reilianum*) [3],[4]. However, there are also non-pathogenic Ustilaginomycetes, such as *Pseudozyma antarctica* and *Pseudozyma tsukubaensis*.

Although these plant diseases lead to a considerable yield reduction of cereal production, smut fungi also have an economically positive side. They naturally produce a wide range of value-added chemicals (e.g. secondary metabolites, TCA cycle intermediates) with growing biotechnological interest. Reported metabolites are polyols, organic acids, extracellular glycolipids, iron-chelating siderophores and tryptophan derivatives [5],[6]. Polyols, such as erythritol (ery) and mannitol, for example, have large markets as sweeteners for diabetics and as facilitating agents for the transportation of pharmaceuticals in medicine [7],[8]. Itaconic (ita), L-malic (mal), succinic (suc), *l*-itatartaric (itt), and *l*-2-hydroxyparaconic (hp) acid are organic acids produced by many Ustilaginomycetes [6],[9]. Applications for itaconic acid are for example the production of resins, plastics, adhesives, elastomers, coatings, and nowadays itaconate is discussed as a platform chemical in the production of biofuels [10],[11]. Malic acid is used in many food products, primarily as an acidulant [12]. Succinic acid is utilized as a precursor to pharmaceutical ingredients, such as additives, solvents, and polymers, but also as a food additive and dietary supplement [13]. Another category of metabolites produced by smut fungi contains extracellular glycolipids, such as mannosylerythritol lipids (mel) and ustilagic acid (ua) [14]-[16]. These lipids have biosurfactant properties and can be used in pharmaceutical, cosmetic, and food applications and are known for their strong fungicidal activity on many species [5].

Besides the production of this broad range of metabolites Ustilaginaceae have further positive characteristics. The haploid form of many strains grows unicellularly, which is advantageous in comparison to filamentous fungi where control of fungal morphology is an important process determinant [17]. Furthermore, the strains are able to metabolize a variety of poly- and monomers with carbohydrate origin derived from renewable non-food biomass degradation [5],[18],[19], which are the substrates of choice for future biotechnological bulk production processes [20].

These advantages make strains of the family Ustilaginaceae promising candidates for industrial production of polyols, acids and lipids. So far large scale erythritol production by *Pseudozyma tsukubaensis* is the only reported industrial use of Ustilaginaceae, which is also a notable itaconate producer with up to 75 g L^−1^ itaconate [8],[9]. While the fundamental biology of these species is studied in great detail, the biotechnological exploitation is still a relatively unexplored field. The production, as well as the ratio of the different products is strongly influenced by both the chosen strain and culture conditions [6],[21]. To exploit nature’s biosynthetic capabilities, 68 Ustilaginaceae of 13 species were screened for their production of itaconate, malate, succinate, erythritol, ustilagic acid, and mannosylerythritol lipids from glucose to characterize their biodiversity and identify potential novel strains for biocatalysis. High performing strains for the best itaconate and malate production were characterized in more detail in bioreactor experiments.

## Results and discussion

### Screening for best producer of itaconate, malate, succinate, erythritol, ustilagic acid, and mannosylerythritol lipids

**Figure 1:**
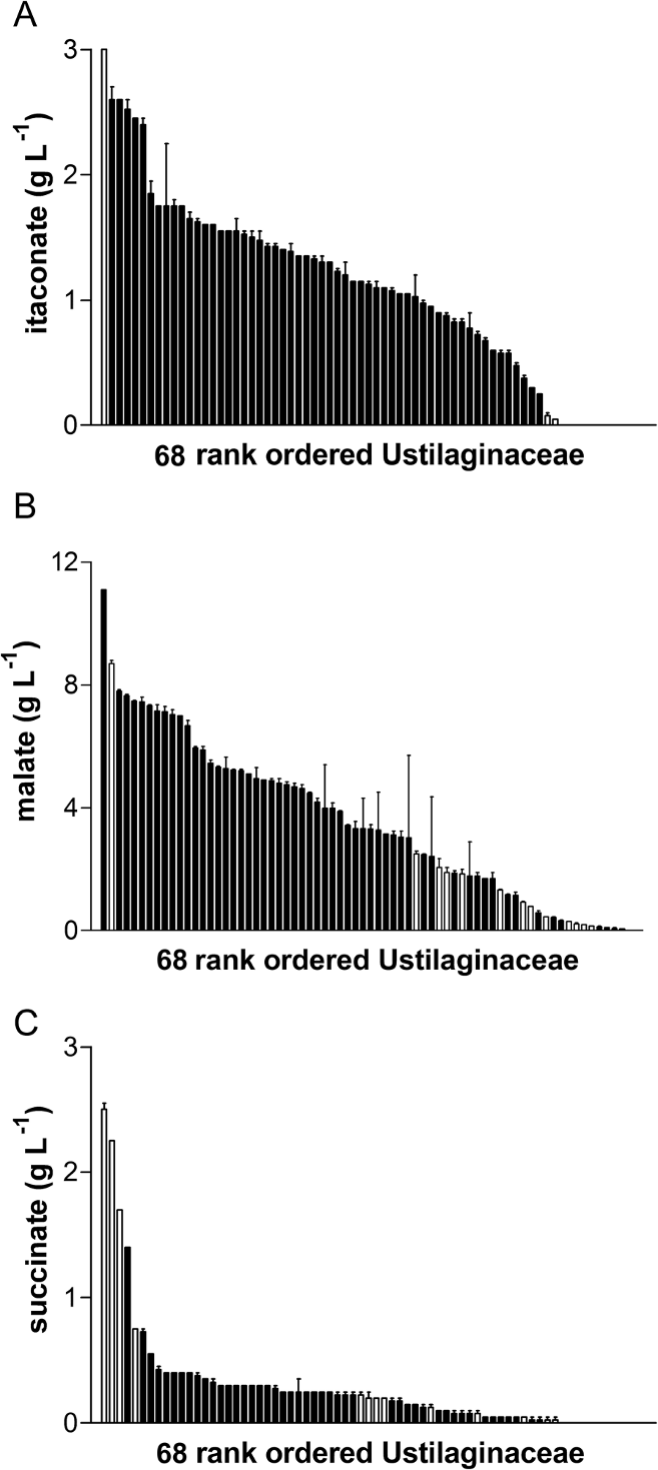
**Overall biodiversity of different Ustilaginaceae.** Concentration of itaconate **(A)**, malate **(B)** and succinate **(C)** produced by different Ustilaginaceae were measured after 96 h (itaconate, succinate) or 48 h (malate) cultivation in CaCO_3_ buffered screening medium. Black bars are *U. maydis* strains, white bars other Ustilaginaceae. The values are the arithmetic mean of two biological determinations. Error bars indicate deviation from the mean. Strain numbers are available in Additional file 1.

For identification of potential novel strains for biocatalysis, 68 Ustilaginaceae of 13 species, with special focus on 56 *U. maydis* strains, were cultivated in two different buffered, defined media and screened for their extracellular production of itaconate, malate, succinate, erythritol, and glycolipids. Table 1 and Figure 1 show an overview of the high biodiversity of products and their amounts. Raw data are provided in Additional file 1.

**Table 1:** **Overview of itaconate (ita), malate (mal), succinate (suc), erythritol (ery), ustilagic acid (ua), and mannosylerythritol lipids (mel)**

	ita	mal	ery	suc	ua ^a^		mel ^a^	
	C	C	C	C	C	M	C	M
***Ustilago maydis*** **2162** ^**b**^	++	++++	-	++	-	*	-	-
***Macalpinomyces eriachnes*** **2209**	-	+++	-	++	-	*	**	*
***Sporisorium consanguineum*** **2210**	-	+	+	++	-	-	-	-
***Sporisorium cruentum*** **2211**	-	++++	+	+	-	-	**	***
***Sporisorium exsertum*** **2212**	-	++	+++	+	-	-	*/-	*/-
***Sporisorium scitamineum*** **2213**	-	++	++	+	-	***	-	***
***Sporisorium walkeri*** **2214**	-	++	+	+	-	*/-	-	-
***Ustanciosporium gigantosporum*** **2215**	-	+	-	-	-	-	-	*
***Ustilago avenae*** **2216**	-	+	-	++	-	-	-	-
***Ustilago cynodontis*** **2217**	+++	+	++	+	-	-	-	-
***Ustilago filiformis*** **2218**	-	++	+	+	-	-	-	-
***Ustilago vetiveriae*** **2220**	+	++	-	+	-	-	-	-
***Ustilago xerochloae*** **2221**	+	+	+	+	-	-	-	-

Chemicals produced by several Ustilaginaceae after 96 h (malate after 48 h) of cultivation in screening media buffered with 100 mM MES (M) or 33 g L^−1^ CaCO_3_ (C). (− = no production, + = < 1 g L^−1^, ++ = 1–3 g L^−1^, +++ = 3–6 g L^−1^, ++++ = > 6 g L^−1^, */- = no/low lipid production, * = low lipid production, ** = lipid production, *** = high lipid production).

Strain numbers correspond to Additional file 1.

^a^no concentrations, relative ratio estimated from TLC.

^b^ *Ustilago maydis* 2162 was chosen as the best producer of itaconate, malate and succinate among all tested *U. maydis* strains.

In the first CaCO_3_ buffered screening the strains showed a high variety of products and their amounts. Among the species *U. maydis,* the acid concentration differed highly, although the different strains generally produced the same products (Additional file 1, Figure 1). However, there were strains which stood out by their relatively high acid production. *U. cynodontis* 2217 was identified as the best itaconate producer with 3.3 ± 0.1 g L^−1^ itaconate and a yield of 0.1 ± 0.0 g_ita_ g_glc_^−1^ (Figure 1A). The best malate producer was *U. maydis* 2162 with 11.1 ± 0.0 g L^−1^ malate and a yield of 0.3 ± 0.0 g_mal_ g_glc_^−1^ after 48 h cultivation (Figure 1B). The final malate titer of *S. cruentum* 2211 was in the same range with 11.0 ± 0.0 g L^−1^ and a yield of 0.3 ± 0.0 g_mal_ g_glc_^−1^. However, the production rate was lower, because the highest malate titer was reached after 96 h. Therefore, *U. maydis* 2162 was the preferred malate producer. The highest succinate concentration of 2.5 ± 0.1 g L^−1^ was reached by *U. avenae* 2216 corresponding to a yield of 0.1 ± 0.0 g_suc_ g_glc_^−1^ (Figure 1C). *S. exsertum* 2212 produced the highest amount of erythritol, namely 3.8 ± 0.0 g L^−1^ with a yield of 0.1 ± 0.0 g_ery_ g_glc_^−1^ (Additional file 1, ery).

In addition, the variety and relative ratio of produced (glyco-) lipids amongst the tested genera and species differed considerably (Table 1). Because of the high variety of possible lipids for which no standards are available, the substances cannot be identified and quantified exactly. Therefore, the relative ratio was estimated by visual inspection of TLCs.

An additional screening in MES buffered screening medium was performed. MES is known for its significant impact on fungal metabolism [22],[23]. Therefore, the influence of MES on growth and acid production of several representative strains of the family of Ustilaginaceae, was investigated using cultures with MES, CaCO_3_, and a combination of both buffers. Neither growth nor acid production were negatively influenced by the presence of MES per se (data not shown). Therefore, MES was considered to be a suitable cultivation buffer. The MES buffered screening confirmed the best producer for itaconate, malate, and succinate (Additional file 1), although overall concentrations were generally lower than in CaCO_3_ buffered medium. Furthermore, the different buffer agents had a significant impact, especially on the mannosylerythritol lipids and ustilagic acid production. Additional file 1 (TLC) shows the TLC of extracted lipids produced by different Ustilaginaceae cultivated for 96 h in screening medium buffered with MES or CaCO_3_ exemplarily for 12 different strains. In cultivations with the stronger CaCO_3_ buffer none of the strains produced ustilagic acid and also less mannosylerythritol lipids were produced compared to the MES buffered cultivation (Additional file 1, TLC), indicating that extracellular lipids were preferentially produced at low pH values. An analytical effect of CaCO_3_ related to the precipitation of calcium salts of ustilagic acid was excluded by addition of CaCO_3_ to samples of MES buffered cultivations after 96 h resulting in the same TLC banding pattern as those of standard MES buffered cultivations (Additional file 1, TLC control). A similar pH sensitivity was already shown for the mannosylerythritol lipid production of *Candida* sp. strain SY16 using pH controlled fed-batch fermentations [24].

The screening confirmed the potential of the Ustilaginaceae to produce a wide range of biotechnologically interesting chemicals, if the selected strains are further optimized. After optimization they may compete with already published, better producing wildtype strains [6],[8],[25].

An optimization strategy to find the best suitable candidate among the family Ustilaginaceae for industrial production of organic acid, such as itaconate, malate, and succinate, should contain the following four steps. First a species amongst the Ustilaginaceae needs to be identified, which produces the desired product as the main product with high yield. In the screening approach the different species showed a high variety of product combinations. However, there is still a huge biodiversity even among one species, such as *Ustilago maydis*. The results showed that the product spectrum between the *U. maydis* strains remained the same, whereas the concentrations of these products varied considerably, for instance 97% between the best and the worst itaconate producer. This leads to step two, which is the screening for an optimal producer of the desired product amongst one species covering a significant number of strains. Thirdly, the chosen strain can be further improved by metabolic engineering. Engineering of the strain may focus on the deletion of metabolic reactions leading to by-products, since for an industrial acid production the product spectrum of *U. maydis* is still too versatile. The most ideal outcome is one product with a high rate, yield, and titer preferably without any by-product [26]. First attempts have already been made by deleting the genes *emt1* (*um03117*) and *cyp1* (*um11812*) of *U. maydis* to disrupt the production of mannosylerythritol lipids and ustilagic acid [14],[27]. Investigation of these knockouts in a biotechnological context would be a promising approach. Another engineering technique is the up-regulation of pathways, which leads to the desired product - providing that these are known - for example by integration of stronger promoters to get higher expression of the involved genes [28]. The development of efficient genetic tools especially for *U. maydis* greatly facilitates these metabolic engineering efforts [29]. The last step is the optimization of the cultivation conditions for the product of choice. In former studies, the optimization of the cultivation process for itaconate with *Aspergillus terreus* for example successfully resulted in an increased production [10],[30]-[39]. In the end this strategy leads to an optimized strain and process, engineered for one specific product, with high potential for industrial applications delivering contributions to a sustainable bioeconomy.

### pH dependency

Given the different results obtained in both screening buffers, the influence of pH on the metabolite production was further investigated. These investigations were performed in MES buffered screening media (pH 6.5) with differing MES concentrations in a range from 30 mM to 100 mM. For this, the strains *U. maydis* 2162, *U. maydis* 2229, and *U. cynodontis* 2217 were chosen due to their high malate and itaconate production in the prior analysis. MES buffered cultures experience a gradual pH decrease as acids are produced, which continues until the pH minimum for acid production is reached. The rate of this decrease depends on the MES concentration. Therefore, the pH at the end of the culture, along with the dependency of the final acid concentration on the MES concentration, might be considered as a reflection of the pH minimum for acidic product formation.

**Figure 2:**
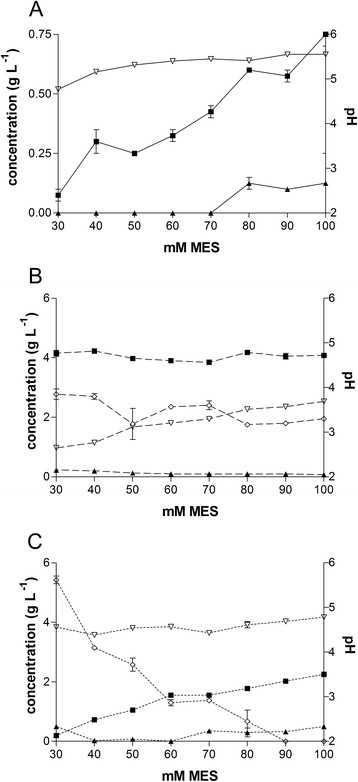
**The influence of the buffer concentration on the acid production.** pH (▽) and concentration of itaconate (■), malate (▲) and erythritol (◇) produced by **A**: *U. maydis* 2229 (solid lines) and **B**: *U. cynodontis* 2217 (dashed lines) after 120 h and **C**: *U. maydis* 2162 (dotted lines) after 72 h cultivation in screening medium containing different MES concentrations. The values are the arithmetic mean of two biological determinations. Error bars indicate deviation from the mean.

The final pH after cultivation differed greatly between the three strains, while the different buffer concentrations had a much lower impact (Figure 2). The final pH of *U. maydis* 2229 was 5.3 ± 0.2, much higher than the final pH of *U. maydis* 2162 with 4.6 ± 0.1 and *U. cynodontis* 2217 with 3.3 ± 0.5, indicating a higher pH minimum of *U. maydis* 2229 and a lower pH limit for acid production of *U. cynodontis*. Generally, in CaCO_3_ buffered cultivations (Figure 1) or pH-controlled batch fermentations (below) the produced acid concentrations were higher in comparison to them presented in Figure 2, confirming the general assumption that in most strains acid production is limited by the MES concentration.

Additionally, Figure 2 shows also the pH dependency of the itaconate, malate, succinate, mannosylerythritol lipids, and ustilagic acid production. *U. maydis* 2229 and *U. maydis* 2162 produced less itaconate and malate with decreasing buffer concentrations. Interestingly, the acid and erythritol production of *U. cynodontis* 2217 was entirely independent from the buffer concentration and hence from the pH value, indicating that the pH minimum is below the lowest measured value. A higher buffer capacity/concentration increases the titer of acids, and generally an excess of CaCO_3_ is used in shake flasks leading to a final pH ranging from 4 to 5 depending on the buffer concentration [6],[21],[40],[41]. Assuming that the buffer influences the acid production, it is reasonable to investigate other buffers, such as MES, to consider the individual pH minimum/optimum of each strain. Furthermore, this variation has to be confirmed by pH controlled batch fermentations. The erythritol production of *U. maydis* 2162 increased with decreasing buffer concentration, which was also ascertained by Guevarra and Tabuchi for *U. cynodontis* K320 in unbuffered media [40]. This metabolic change from acid production to polyole production is a common protective mechanism for microorganisms in case of pH stress [42].

The different minima can affect the upstream processes of the desired product. Depending on the upstream process conditions the best fitting and most suitable strain out of the broad spectrum can be chosen. For example, for simultaneous saccharification and fermentation (SSF) a pH of approximately 4.8 is preferred due to the pH optima of the used cellulases [21],[43]. This pH prerequisite would apply more for *U. maydis* 2229 or *U. maydis* 2162, whereas *U. cynodontis* 2217 would be better for acid production in batch fermentations. Its low pH minimum facilitates the downstream processing because less base has to be added to back-titrate the stoichiometric amounts of produced acids, decreasing waste and costs, and therefore increasing the value.

The (glyco-) lipid production of *U. maydis* 2229 and *U. maydis* 2162 was also dependent on the buffer concentration (Table 2). With decreasing buffer concentration, *U. maydis* 2229 produced less mannosylerythritol lipids, *U. maydis* 2162 more. Also the ustilagic acid production of *U. maydis* 2229 increased with decreasing buffer concentration, whereas the ustilagic acid production of *U. maydis* 2162 remained constant.

**Table 2:** **Overview of mannosylerythritol lipids (mel) and ustilagic acid (ua) production by selected Ustilaginaceae**

MES (mM)	***U. maydis*** 2229		***U. maydis*** 2162		***U. cynodontis*** 2217	
	mel	ua	mel	ua	mel	ua
**100**	**	-	*	*	-	-
**90**	**	-	*	*	-	-
**80**	**	-	**	*	-	-
**70**	**	-	**	*	-	-
**60**	*	*	**	*	-	-
**50**	*	*	**	*	-	-
**40**	*	*	**	*	-	-
**30**	*	*	**	*	-	-

Samles were taken after 120 h of cultivation in screening media buffered with varying MES concentrations. (relative ratio estimated from TLC, − = no lipid production,* = low lipid production, ** = lipid production).

### Controlled batch fermentation of the best itaconate and malate producer

**Figure 3:**
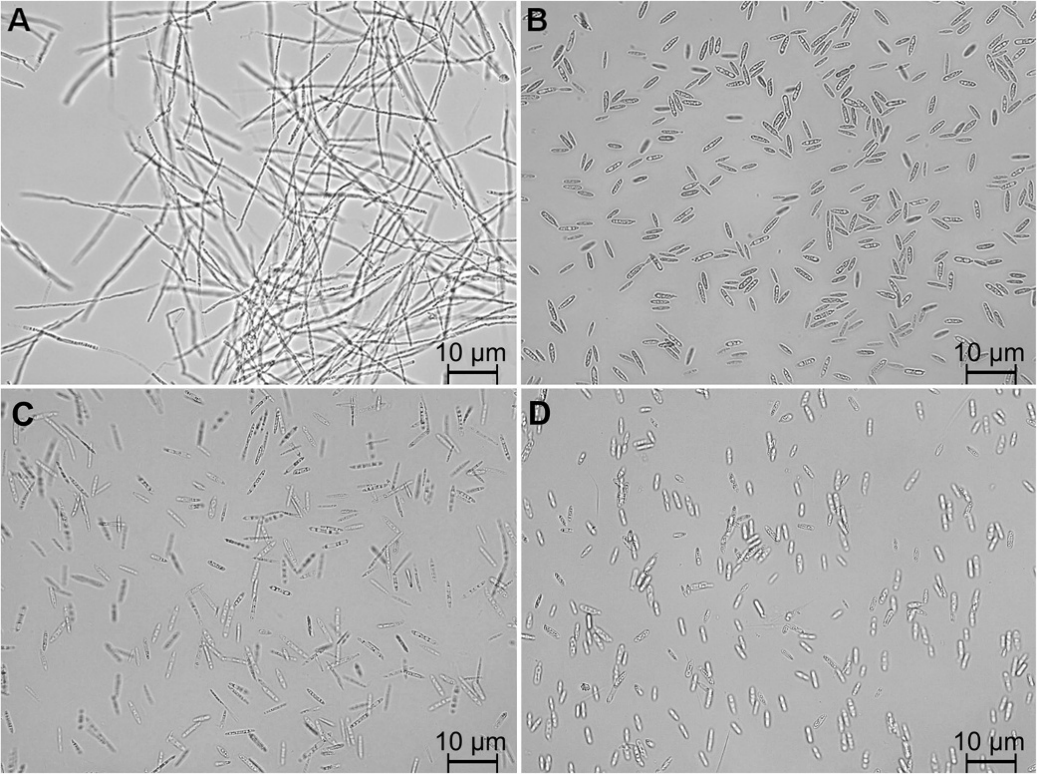
**Light microscopy images of** ***U. cynodontis*** **2217 (A, C) and** ***U. maydis*** **2162 (B, D) cells. A** and **B**: controlled batch fermentation in a bioreactor containing batch medium (200 g L^−1^ glucose, 4 g L^−1^ NH_4_Cl, 30°C, 80% DOT, at pH 6.0). **C** and **D**: System Duetz cultivations containing screening medium (45.5 g L^−1^ glucose, 0.8 g L^−1^ NH_4_Cl, 33 g L^−1^ CaCO_3_, 30°C, 80% DOT, magnification 400×).

Although the buffer has a strong influence on the acid production, for industrial processes, additional parameters have to be investigated, preferably in controlled batch fermentations. To further characterize the production potential of Ustilaginaceae, two strains were chosen based on the following criteria: unicellular growth, best itaconate production, and best malate production. Even though *U. cynodontis* 2217 produced the highest amount of itaconate in the screening approaches in both CaCO_3_ and MES buffer, the strain was not used for further experiments because it displayed strong filamentous growth in stirred tank reactors, in comparison to single cell growth in System Duetz cultivations (Figure 3A and C). The growth of the filamentous fungus on fermenter equipment, such as oxygen and pH electrodes, makes it inexpedient during batch fermentations and can pose additional costs [17]. So far it is still unclear what caused the change from unicellular to filamentous growth between shake-flask and stirred tank reactor growth. Possible causes include differences in shear forces caused by stirring and sparging, different oxygen supply and the presence of antifoam in the bioreactor. *U. cynodontis*’ changing morphology depending on cultivation conditions was also observed by Zapata-Morín et al. [44] and Durieu-Trautmann et al. [45].

**Figure 4:**
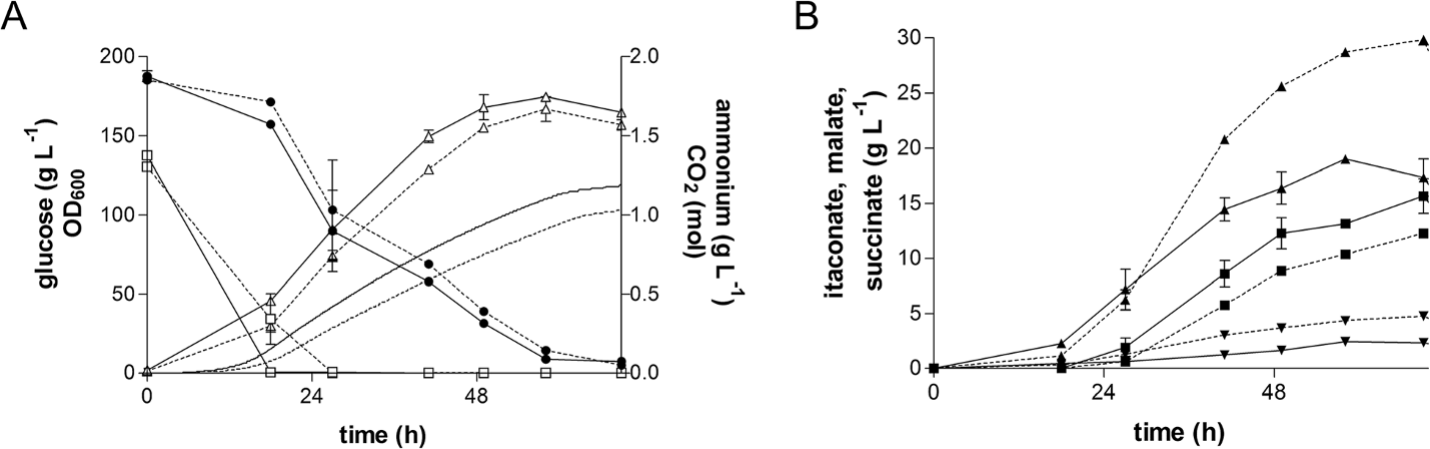
**Controlled batch fermentation of the best itaconate and malate producers. A**: OD_600_ (Δ), emitted CO_2_ amount (without symbols), concentration of glucose (●) and ammonium (□) and **B**: concentration of itaconate (■), malate (▲) and succinate (▼) during fermentation in a bioreactor containing batch medium (200 g L^−1^ glucose, 4 g L^−1^ NH_4_Cl, 30°C, 80% DOT, at pH 6.0) with *U. maydis* 2229 (solid lines) and *U. maydis* 2162 (dotted lines). The values are the arithmetic mean of two biological determinations. Error bars indicate deviation from the mean. For the CO_2_ values, all deviations from the means were under 10%.

Therefore, the two best performing under all cultivation conditions unicellular growing strains *U. maydis* 2229 and *U. maydis* 2162 were cultivated in controlled batch fermentation (Figure 3B and D). The nitrogen source in the *U. maydis* 2229 fermentation was exhausted after 18 h (Figure 4A), although further growth was observed after N depletion up to 58 h. This phenomenon was previously seen and was related to intracellular lipid formation leading to swollen cells, and utilization of internal nitrogen pools for further reproduction cycles [21]. After 58 h the maximal cell dry weight (CDW) of 67 ± 0 g L^−1^ was reached. In comparison to *U. maydis* 2229 the growth phase of *U. maydis* 2162 was longer, with depletion of the N source after 27 h. After 58 h the maximal CDW of 62 ± 0 g L^−1^ was observed. Glucose was almost completely consumed at the end of both fermentations.

After nitrogen depletion, both strains produced a mix of itaconate, malate, and succinate (Figure 4B) as well as traces of an unknown product. The total acid concentrations were 35 ± 4 g L^−1^ for *U. maydis* 2229 and 47 ± 1 g L^−1^ for *U. maydis* 2162. The strain *U. maydis* 2162 is the better malate producer and produced additionally the highest amount of succinate compared to *U. maydis* 2229. However, the strain *U. maydis* 2229 produced the highest amount of itaconate in comparison to *U. maydis* 2162. All production parameters are summarized in Table 3. The addition of extra 100 g L^−1^ glucose at 72 h did not have a significant impact on acid production (data not shown).

**Table 3:** **Production parameters of** ***U. maydis*** **2229 and** ***U. maydis*** **2162 bioreactor fermentations**

		***U. maydis*** 2229	***U. maydis*** 2162
**ita**	**titer** (g L^−1^)	15.7 ± 1.6	12.2 ± 0.4
	**r**_**p, max**_ (g L^−1^ h^−1^)^c^	0.48 ± 0.03	0.38 ± 0.02
	**Y**_**P/S**_ (g_ita_ g_glc_^−1^)^d^	0.08 ± 0.01	0.07 ± 0.00
**mal**	**titer** (g L^−1^)	17.3 ± 1.7	29.9 ± 0.5
	**r**_**p, max**_ (g L^−1^ h^−1^)^c^	0.52 ± 0.21	1.04 ± 0.09
	**Y**_**P/S**_ (g_mal_ g_glc_^−1^)^d^	0.09 ± 0.01	0.16 ± 0.00
**suc**	**titer** (g L^−1^)	2.6 ± 0.4	4.8 ± 0.1
	**r**_**p, max**_ (g L^−1^ h^−1^)^c^	0.04 ± 0.02	0.13 ± 0.03
	**Y**_**P/S**_ (g_suc_ g_glc_^−1^)^d^	0.01 ± 0.00	0.03 ± 0.00
**Y**_**P/S**_ (g_acid_ g_glc_^−1^) ^e^		0.19 ± 0.01	0.25 ± 0.01
**Y**_**X/S**_ (g_biomass_ g_glc_^−1^)^f^		0.36 ± 0.01	0.35 ± 0.02

Fermentation took place in batch medium containing 200 g L^−1^ glucose and 4 g L^−1^ NH_4_Cl, 30°C, 80% DOT, at pH 6.0.

Itaconate (ita), succinate (suc), malate (mal), glucose (glc). The values are the arithmetic mean of two biological determinations. Errors indicate deviation from the mean.

^c^r_p, max_: maximum production rate.

^d^Y_P/S_: yield product per consumed glucose.

^e^Y_P/S_: yield total acid per consumed glucose.

^f^Y_X/S_: yield biomass per consumed glucose.

The batch fermentations could confirm the product range found in the screening approaches, although published itaconate concentrations of 20–45 g L^−1^ produced by *U. maydis* 2229 under similar conditions could not be reached [46],[47]. However, the obtained succinate concentration was in the range of published titers of 5 g L^−1^ produced by different *Ustilago* species [6]. For malate even higher concentrations were reached in comparison to the highest published concentrations of 20 g L^−1^ produced by *U. maydis* [6]. The maximal theoretical yields from glucose for itaconate, malate, and succinate are 0.7 g_ita_ g_glc_^−1^, 1.5 g_mal_ g_glc_^−1^, and 1.3 g_suc_ g_glc_^−1^, respectively, assuming zero growth. These values are still far away from the measured values obtained in this study, indicating that there is still room for further optimization as mentioned in the previous section.

High formation of mannosylerythritol lipids, but no ustilagic acid, was also observed via TLC (data not shown). Additionally, both strains accumulated an unknown product during fermentation with an HPLC retention time of 12.4 min and an UV/RI area ratio of 4.5 ± 0.3 mAU mV^−1^. Guevarra and Tabuchi proposed the appearance of *l*-itatartarate and *l*-2-hydroxyparaconate during itaconate production [40], with relative retention times to itaconate of t_R(itt)_/t_R(ita)_ = 0.64 and t_R(hp)_/t_R(ita)_ = 0.78, respectively, measured by RI detector. The unknown product accumulated by both *U. maydis* strains during batch fermentations could possibly be *l*-2-hydroxyparaconate, since it has a relative retention time to itaconate of t_R(unknown)_/t_R(ita)_ = 0.76. However, this is yet to be confirmed since no standards are commercially available. *U. maydis* 2229 produced nearly twice as much of this unknown compound as *U. maydis* 2162 based on HPLC peak area using the RI detector (data not shown). Mass balancing accounted for 94.1 ± 4.3% (*U. maydis* 2229) and 93.8 ± 0.9% (*U. maydis* 2162) of the added carbon source, indicating that 0.19 ± 0.14 Cmol and 0.19 ± 0.03 Cmol, respectively, were still unaccounted. This unaccounted fraction likely consists of (glyco-) lipids and/or the unknown product observed by HPLC. The unaccounted 0.19 Cmol would correspond to approximately 11.7 g L^−1^ *l*-2-hydroxyparaconate, 13.1 g L^−1^ *l*-itatartarate, 6.8 g L^−1^ mannosylerythritol lipids or 8.3 g L^−1^ ustilagic acid or a mix of these products.

## Conclusions

In summary this study demonstrates the potential of Ustilaginaceae for the production of value-added chemicals. From 68 Ustilaginaceae of 13 species, potential strains with favorable characteristics for the production of itaconate, malate, succinate, and erythritol from glucose were identified. The strains produced a broad range of products with varying concentrations showing the high biodiversity of this microbial family. Besides the product diversity there is also a variation in the pH minimum between the different strains and products, which can be exploited for alternative up- and downstream processes. In the future, this variation has to be confirmed by pH controlled batch fermentations. Furthermore, batch fermentations confirmed the product range found in the screening approaches and highlighted the importance of medium/culture optimization, which combines the previously published biodiversity with new biotechnological aspects. Since Ustilaginaceae are able to use pentoses, such as xylose, which are components of non-food renewable biomass similar screening experiments on these carbon sources might also be conducted in the future. This high potential concerning the broad product spectrum of metabolites together with a unicellular growth pattern and the ability to utilize non-food renewable biomass as carbon source makes the Ustilaginaceae a promising family for the production of valuable chemicals.

## Methods

### Strains, media, and growth conditions

68 strains of the family Ustilaginaceae were used in this study (Additional file 1, strains). Numbers behind the species name indicate the strain number.

Screenings were performed in the System Duetz® (24 well plates) with a filling volume of 1.5 mL (shaking diameter = 50 mm, agitation speed = 300 rpm, temperature = 30°C, and relative air humidity = 80%) [48]. The screening medium contained 45.5 g L^−1^ glucose, 0.8 g L^−1^ NH_4_Cl, 0.2 g L^−1^ MgSO_4_·7H_2_O, 0.01 g L^−1^ FeSO_4_·7H_2_O, 0.5 g L^−1^ KH_2_PO_4_, 1 mL L^−1^ vitamin solution, 10 mL L^−1^ trace element solution, and as buffer 33 g L^−1^ calcium carbonate (CaCO_3_) or 19.5 g L^−1^ 2-(N-morpholino)ethanesulfonic acid (MES). The pH of the MES stock solution was adjusted to 6.5 with NaOH. The vitamin solution contained (per liter) 0.05 g D-biotin, 1 g D-calcium panthotenate, 1 g nicotinic acid, 25 g myo-inositol, 1 g thiamine hydrochloride, 1 g pyridoxol hydrochloride, and 0.2 g para-aminobenzoic acid. The trace element solution contained (per liter) 1.5 g EDTA, 0.45 g ZnSO_4_·7H_2_O, 0.10 g MnCl_2_·4H_2_O, 0.03 g CoCl_2_·6H_2_O, 0.03 g CuSO_4_·5H_2_O, 0.04 g Na_2_MoO_4_·2H_2_O, 0.45 g CaCl_2_·2H_2_O, 0.3 g FeSO_4_·7H_2_O, 0.10 g H_3_BO_3_, and 0.01 g KI. Samples were taken after 48 h and 96 h, since initial experiments showed that the highest concentration of malate is reached by most of the strains after 48 h and carbon source is completely depleted after 96 h (data not shown).

Batch cultivations were performed in a New Brunswick BioFlo® 115 bioreactor (Eppendorf, Germany) with a total filling volume of 1.3 L and a working volume of 0.5 L. Cultivation conditions were chosen according to Maassen et al. [46]. All cultivations were performed in batch medium containing 200 g L^−1^ glucose, 4 g L^−1^ NH_4_Cl, 0.2 g L^−1^ MgSO_4_·7H_2_O, 0.01 g L^−1^ FeSO_4_·7H_2_O, 0.5 g L^−1^ KH_2_PO_4_, 1 g L^−1^ yeast extract (Merck Millipore, Germany), 1 mL L^−1^ vitamin solution, and 10 ml L^−1^ trace element solution. During cultivation, pH 6.0 was maintained by automatic addition of 10 M NaOH, and the dissolved oxygen tension (DOT) was kept constant above approximately 80% saturation by automatic adjustment of the stirring rate (700–1200 rpm). The bioreactor was aerated at a rate of 1 L min^−1^ (2 vvm). The temperature was set at 30°C. Level sensor controlled antifoam 204 (Sigma Life Science, USA) was added to prevent foam formation. The bioreactor was inoculated to a final OD_600_ of 1.5 with cells from an overnight culture in 50 mL screening medium, which were washed two times with 0.9% NaCl. Bioreactor off-gas analysis for online monitoring of CO_2_ and O_2_ content were performed with BlueInOne_Ferm_ off-gas sensors (BlueSens gas sensor GmbH). The online CO_2_ signal (%) was converted into the absolute emitted CO_2_ amount in mol by multiplying the gas flow rate (L min^−1^) with the CO_2_ content (%) and the molar volume of an ideal gas at 1 atmosphere of pressure and 25°C (L mol^−1^). Mass balancing was achieved by subtracting the carbon amount of biomass, off-gas, and products, such as itaconate, malate, and succinate, from the substrate glucose.

### Analytical methods

All values are the arithmetic mean of two biological determinations. Error bars or ± −values indicate the deviation from the mean for two values.

Cell densities were measured by determining the absorption at 600 nm with a Unico spectrophotometer 1201.

For dry weight determination 3 mL culture broth was filtered using Macherey-Nagel Paper MN218B (Macherey-Nagel, Germany) and weighed after drying at 110°C for 24 h.

Light microscopy images were taken with a Leica DM750 microscope with 400× magnification and a Leica ICC50 camera (Leica Microsystems GmbH, Wetzlar, Germany).

Glucose, itaconate, malate, succinate, and erythritol in the supernatants were analyzed in a Beckmann Coulter System Gold High Performance Liquid Chromatography (Beckmann Coulter GmbH, Germany) with an Organic Acid Resin 300 × 8 mm column (CS-Chromatography, Germany) and a differential refractometer LCD 201 (MELZ, Germany) or an UV detector Beckmann Coulter System Gold 166 Detector (210 nm, 5 Hz) (Beckmann Coulter GmbH, Germany). As solvent, 5 mM H_2_SO_4_, with a flow rate of 0.6 mL min^−1^ and a temperature of 30°C, was used. All samples were filtered with Rotilabo® syringe filters (CA, 0.20 μm, Ø 15 mm) and afterwards 1:5 diluted with 5 mM H_2_SO_4_.

The ammonium concentration in the culture supernatant was measured by a colorimetric method according to Willis using salicylate and nitroprusside [49].

Glycolipids, such as mannosylerythritol lipids and ustilagic acid, were analyzed by thin-layer chromatography (TLC). Therefore, 0.6 mL ethyl acetate were added to 0.3 mL culture and mixed for 20 min. After centrifugation (14,000 g, 5 min) the ethyl acetate phase was dried overnight at 70°C and resuspended in 50 μL methanol. 5 μL of extract were spotted on a TLC Silica gel 60 aluminum plate (20 cm × 20 cm, Merck KGaA, Darmstadt Germany). A running buffer comprising 71% (v/v) chloroform, 28% (v/v) methanol, and 1% (v/v) H_2_O was used. For staining, the plate was sprayed with a mix of 97% (v/v) acetic acid, 1% (v/v) *p*-anisaldehyde, and 2% (v/v) H_2_SO_4_ solution followed by 20 min of heating at 120°C. All spots with a retardation factor (R_f_ = migration distance of the substance/migration distance of the solvent front) smaller than 0.62 were defined as ustilagic acid, everything else as mannosylerythritol lipids [50]. Brightness, color saturation, and contrast were modified in the TLC pictures to enhance visibility.

## Additional file

## Electronic supplementary material

Additional file 1: **Provides the strain list and all raw data of the screenings in MES and in CaCO**_**3**_ **buffer including concentrations of itaconate, malate, erythritol, succinate, and relative amounts of mannosylerythritol lipids, ustilagic acid, and the unknown product.** Additionally, an examplary TLC of extracted lipids produced by different Ustilaginaceae including an control TLC is provided. (XLS 5 MB)

Below are the links to the authors’ original submitted files for images.

Authors’ original file for figure 1

Authors’ original file for figure 2

Authors’ original file for figure 3

Authors’ original file for figure 4
