# Supplementary figures and images for: Prospecting the biodiversity of the fungal family Ustilaginaceae for the production of value-added chemicals
Source: Fungal Biol Biotechnol. 2014 Nov 1;1:2. doi: 10.1186/s40694-014-0002-y (PMC5598272; doi:10.1186/s40694-014-0002-y)

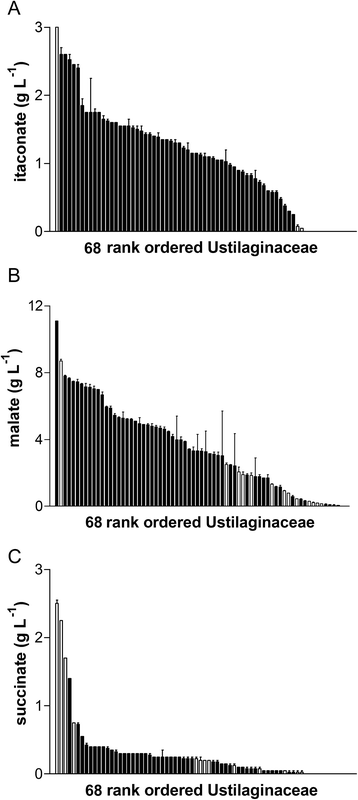

Supplement: Supplementary file 2 — Authors’ original file for figure 1 [file 40694_2014_2_MOESM2_ESM.gif]

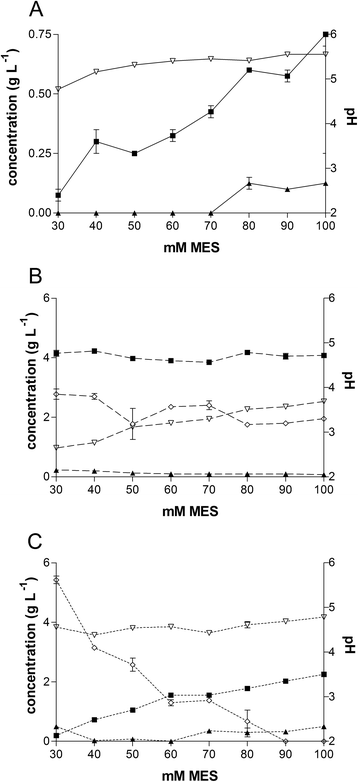

Supplement: Supplementary file 3 — Authors’ original file for figure 2 [file 40694_2014_2_MOESM3_ESM.gif]

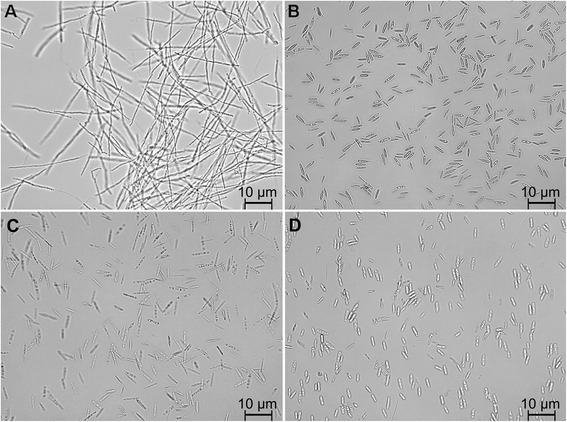

Supplement: Supplementary file 4 — Authors’ original file for figure 3 [file 40694_2014_2_MOESM4_ESM.gif]

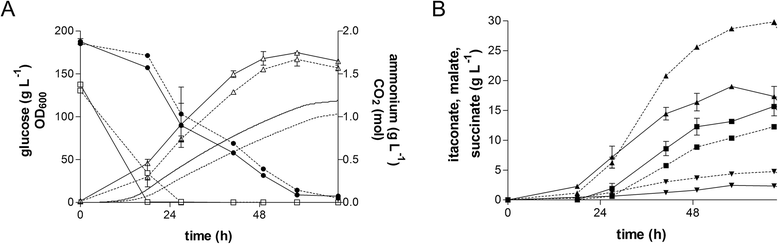

Supplement: Supplementary file 5 — Authors’ original file for figure 4 [file 40694_2014_2_MOESM5_ESM.gif]
